# Association between epigenetic age and type 2 diabetes mellitus or glycemic traits: A longitudinal twin study

**DOI:** 10.1111/acel.14175

**Published:** 2024-04-25

**Authors:** Ke Miao, Xuanming Hong, Weihua Cao, Jun Lv, Canqing Yu, Tao Huang, Dianjianyi Sun, Chunxiao Liao, Yuanjie Pang, Runhua Hu, Zengchang Pang, Min Yu, Hua Wang, Xianping Wu, Yu Liu, Wenjing Gao, Liming Li

**Affiliations:** ^1^ Department of Epidemiology and Biostatistics, School of Public Health Peking University Beijing China; ^2^ Key Laboratory of Epidemiology of Major Diseases (Peking University) Ministry of Education Beijing China; ^3^ Qingdao Center for Disease Control and Prevention Qingdao China; ^4^ Zhejiang Center for Disease Control and Prevention Hangzhou China; ^5^ Jiangsu Center for Disease Control and Prevention Nanjing China; ^6^ Sichuan Center for Disease Control and Prevention Chengdu China; ^7^ Heilongjiang Center for Disease Control and Prevention Harbin China

**Keywords:** aging, epigenetic clock, glycemic traits, twins, type 2 diabetes mellitus

## Abstract

Epigenetic clocks based on DNA methylation have been known as biomarkers of aging, including principal component (PC) clocks representing the degree of aging and DunedinPACE representing the pace of aging. Prior studies have shown the associations between epigenetic aging and T2DM, but the results vary by epigenetic age metrics and people. This study explored the associations between epigenetic age metrics and T2DM or glycemic traits, based on 1070 twins (535 twin pairs) from the Chinese National Twin Registry. It also explored the temporal relationships of epigenetic age metrics and glycemic traits in 314 twins (157 twin pairs) who participated in baseline and follow‐up visits after a mean of 4.6 years. DNA methylation data were used to calculate epigenetic age metrics, including PCGrimAge acceleration (PCGrimAA), PCPhenoAge acceleration (PCPhenoAA), DunedinPACE, and the longitudinal change rate of PCGrimAge/PCPhenoAge. Mixed‐effects and cross‐lagged modelling assessed the cross‐sectional and temporal relationships between epigenetic age metrics and T2DM or glycemic traits, respectively. In the cross‐sectional analysis, positive associations were identified between DunedinPACE and glycemic traits, as well as between PCPhenoAA and fasting plasma glucose, which may be not confounded by shared genetic factors. Cross‐lagged models revealed that glycemic traits (fasting plasma glucose, HbA1c, and TyG index) preceded DunedinPACE increases, and TyG index preceded PCGrimAA increases. Glycemic traits are positively associated with epigenetic age metrics, especially DunedinPACE. Glycemic traits preceded the increases in DunedinPACE and PCGrimAA. Lowering the levels of glycemic traits may reduce DunedinPACE and PCGrimAA, thereby mitigating age‐related comorbidities.

## Graphical abstract

Glycemic traits are positively associated with epigenetic age metrics, especially DunedinPACE. Glycemic traits preceded the increases in DunedinPACE and PCGrimAA. Lowering the levels of glycemic traits may reduce epigenetic clocks, thereby mitigating age‐related comorbidities.

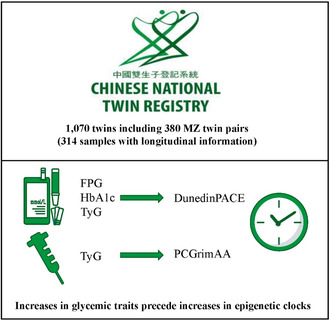

**Abbreviations**
BMI: Body Mass Index
CFI: Comparative fit index
CI: Confidence interval
CNTR: Chinese National Twin Registry
DNAm: DNA methylation
DRCGEE: Doubly robust conditional generalized estimating equations
DZ: Dizygosity twin
EAA: Epigenetic age acceleration
FPG: Fasting plasma glucose
GrimAA: GrimAge acceleration
HbA1c: Hemoglobin A1c
ICC: Intraclass correlations
IPAQ: International Physical Activity Questionnaire
MZ: Monozygosity twin
PC: Principal components
PCGrimAA: PCGrimAge acceleration
PCPhenoAA: PCPhenoAge acceleration
PhenoAA: PhenoAge acceleration
SNP: Single nucleotide polymorphism
SRMR: Standard root mean square residual
T2DM: Type 2 diabetes mellitus
TyG: Triglyceride glucoseindex

## 1 INTRODUCTION

Type 2 diabetes mellitus (T2DM) is an age‐related disease, and its prevalence and incidence continuously increase with advancing age (Chang et al., 2019; Fang et al., 2020; Kuan et al., 2021). Moreover, T2DM patients are more likely to develop age‐related comorbidities such as cardiovascular disease and Alzheimer's disease (Palmer, Gustafson, et al., 2019). Currently, it is unclear whether T2DM promotes or is caused by aging (Morley, 2008; Palmer, Gustafson, et al., 2019).

Variations in physiological function occur among individuals of the same age due to age‐related biological changes (Jylhava et al., 2017). So, chronological age may not be the most suitable biomarker of aging. Age‐related changes in DNA methylation levels have been observed, with evidence suggesting a strong correlation between chronological age and methylation changes at approximately one‐third of methylation sites from peripheral blood samples (Johansson et al., 2013). Based on this fact, various epigenetic clocks have been developed, and they capture different aspects of aging (Sugden et al., 2022).

GrimAge and PhenoAge, were trained using physiological variables to capture physiological dysregulation in the aging process and were associated with many age‐related diseases (Oblak et al., 2021). The GrimAge clock, including 1030 cytosine‐phosphate‐guanine (CpG) sites, was developed using seven plasma proteins, smoking pack‐years, and chronological age as training phenotypes (Lu et al., 2019). The PhenoAge clock is a set of 513 CpG sites derived from chronological age and nine blood measures as training phenotypes (Levine et al., 2018). However, the majority of individual CpGs obtained from DNA methylation microarrays are unreliable, and the technical noise threatens the reliability of epigenetic clocks (Chen et al., 2016; Sugden et al., 2020). Hence, the principal components (PC) of CpGs instead of individual CpGs were used to construct PC versions of epigenetic clocks, called PC clocks (including PCGrimAge and PCPhenoAge), showing more reliability especially in longitudinal studies (Higgins‐Chen et al., 2022). Another epigenetic clock, called DunedinPACE, is a set of 173 CpG sites associated with the longitudinal change rate of 19 biological markers over 20 years (Belsky et al., 2022). The differences between chronological age and PCGrimAge/PCPhenoAge are called PCGrimAge acceleration (PCGrimAA) and PCPhenoAge acceleration (PCPhenoAA). PCGrimAA and PCPhenoAA represent the degree of aging, and DunedinPACE represents the pace of aging at a single time (Moqri et al., 2023).

Associations of epigenetic age metrics (GrimAA or PhenoAA) with T2DM and glycemic traits have been reported in some (Ammous et al., 2021; Arpon et al., 2019; Lee & Park, 2020; Lo & Lin, 2022; Lundgren et al., 2022; Oblak et al., 2021) but not all (Fraszczyk et al., 2022) research. Only one study focused on DunedinPACE and found that DunedinPACE was positively associated with T2DM (Lin, 2023). Genetic factors affect both epigenetic clocks and T2DM (Jylhava et al., 2019; Zhou et al., 2018); this further introduces the possibility of genetic confounding in the assessment of the associations of epigenetic age metrics with T2DM and glycemic traits. The co‐twin control study can exclude genetic confounding, and the design has been used to explore the true associations of epigenetic age metrics with BMI (Lundgren et al., 2022) and metabolic syndrome (Föhr et al., 2024). Furthermore, to explore the causal mechanisms between epigenetic clocks and T2DM, a Mendelian randomization study found that it is GrimAA rather than PhenoAA casually correlated with T2DM (Kong et al., 2023). However, causal mechanisms between other epigenetic clocks and T2DM/glycemic traits remain unclear so far.

Here, using data from the Chinese National Twin Registry (CNTR), we assessed the associations of three epigenetic age metrics (PCGrimAA, PCPhenoAA, and DunedinPACE) with T2DM and glycemic traits. Not only that but we also conducted a co‐twin control study to evaluate whether genetic factors impact these associations. We also aimed to identify the temporal relationships between epigenetic age metrics and T2DM or glycemic traits using a longitudinal study design. The findings of our study may provide clues for the causality of epigenetic aging and T2DM or glycemic traits.

## 2 METHODS

### 2.1 Study participants

The CNTR is the largest national cohort of twins in China. An overview of the cohort design has been provided previously (Gao et al., 2019). Participants included in this study were from two thematic surveys of CNTR in 2013 and 2017–2018. The CNTR conducted in‐person baseline and follow‐up assessments, with health and lifestyle questionnaires completed and fasting blood samples collected at both time points. All participants signed informed consent forms. Ethical approval was obtained from the Biomedical Ethics Committee at Peking University (IRB00001052‐13022 and IRB00001052‐14021).

The inclusion criteria for participants were outlined below: (1) age 18 years or older, (2) complete questionnaire survey and physical examination, and (3) available blood samples. Pregnant twins were excluded. We further removed incomplete co‐twin pairs if one twin had missing DNAm and phenotypic data. Finally, overall samples of 1070 participants (535 twin pairs, including 380 monozygotic [MZ] and 155 dizygotic [DZ] twin pairs) were included in this study. Among these, 314 participants (157 twin pairs, including 95 MZ and 62 DZ twin pairs) had baseline and follow‐up data.

### 2.2 Measurements of glycemic traits and T2DM

We adopted peripheral blood samples from all participants at least after fasting for 8 h, and sent the blood samples to qualified companies for biochemical measurements. The glycemic traits in this study included fasting plasma glucose (FPG), hemoglobin A1c (HbA1c), and triglyceride glucose (TyG) index, a measure of insulin resistance. The TyG index was defined as $\mathrm{TyG}=\ln\left[\frac{\text{fasting triglyceride}\;\left(\frac{\mathrm{mg}}{\mathrm{dL}}\right)\times \mathrm{FPG}\left(\mathrm{mg}/\mathrm{dL}\right)}{2}\right]$. According to the American Diabetes Association criteria (American Diabetes, 2021), a FPG level ≥7.0 mmol/L or serum HbA1c ≥ 6.5% was considered as diabetes. T2DM was diagnosed in patients over 30 years of age who met one of the aforementioned conditions or were diagnosed in a hospital at or above the district/county level, or were currently using antidiabetic medications.

### 2.3 Measurements of covariates

Based on prior literature, we selected several factors that may influence epigenetic clocks as covariates, including age, sex, BMI, education, lifestyle factors, and hypoglycemic agents (Foster et al., 2023; Horvath et al., 2016; Kankaanpää et al., 2021, 2022; Kresovich et al., 2021; Oblak et al., 2021; Tang et al., 2023; Zhao et al., 2019). Weight and height were measured using standard anthropometers, and BMI was calculated as weight in kilograms divided by height in square meters. Education, smoking, alcohol consumption, physical activity, and the use of hypoglycemic agents were obtained by an interview‐administered questionnaire. The highest level of education was recorded and was recorded into three categories: low (primary school and below), middle (junior middle school or senior high school), and high (junior college and above). Smoking was classified as current, former, or never smoking (J. Liu et al., 2010). Based on the number of pack‐years of smoking, smoking pack‐years were categorized into three groups: never smoked, smoked <30 pack‐years, and smoked ≥30 pack‐years (Ye et al., 2019). Alcohol consumption was classified as current, former, or never drinking (Giraldi et al., 2017). According to the International Physical Activity Questionnaire (IPAQ), physical activity was classified as low, moderate, or high (Rosenbaum, Ward, & International Working, 2016).

### 2.4 DNA methylation data collection and processing

Illumina Human Methylation 450K BeadChip arrays or Illumina Infinium Methylation EPIC (850 K) BeadChip arrays were used to collect DNA methylation data from all peripheral blood samples. Only overlapping CpG sites between 450 k and 850K BeadChips were considered in the subsequent procedure, which were obtained using the “combineArrays” function of the R package *minfi* (1.40.0).

For quality control, we implemented several criteria to exclude probes and samples in our analysis. Probes were removed if they met one or more of the following conditions: (1) had detection *p* > 0.05 in more than 1% of samples; (2) were multihit probes; (3) were annotated single nucleotide polymorphisms (SNPs) on the microarray; and (4) were located on the sex chromosomes. As for sample quality control, we excluded samples that (1) had detection *p* > .01; and (2) exhibited sex mismatch. The DNA methylation level of each CpG site was estimated by the β‐value (number of methylated sites/[number of methylated and unmethylated sites]). The quantile normalization was conducted using the “preprocessQuantile” function of the R package *minfi* (1.40.0).

In addition, we used a set of 59 SNPs on both 450K and 850K BeadChip arrays to calculate the correlation of SNPs within twin pairs. If the correlation coefficients were ≥0.9, the twin pairs were defined as MZ twin pairs; otherwise, they were defined as DZ twin pairs (Wang et al., 2015).

### 2.5 Estimation of epigenetic age metrics

Three epigenetic clocks were calculated in this study, including PCGrimAge, PCPhenoAge, and DunedinPACE. PC clocks used principal components rather than individual CpGs as input in the prediction model, and they were calculated using the R code given by the researchers (Higgins‐Chen et al., 2022). The epigenetic age acceleration (EAA) values of PCGrimAge and PCPhenoAge, called PCGrimAA and PCPhenoAA, were the residuals obtained from regressing the PC clocks on chronological age. The values of PCGrimAA and PCPhenoAA represented the degree of aging. Furthermore, to investigate the longitudinal change in PCGrimAge and PCPhenoAge between two time points, we generated continuous variables (“PCGrimAge rate” and “PCPhenoAge rate”) that represented the rate of PCGrimAge and PCPhenoAge change per calendar year (Kim et al., 2021; Wolf et al., 2019). If the values of the PCGrimAge rate or PCPhenoAge rate are >1, it indicates an accelerated rate of epigenetic age change. As PCGrimAge rate and PCPhenoAge rate, if the value of DunedinPACE is >1, it indicates a faster rate of aging (Belsky et al., 2022). DunedinPACE was calculated using R package *DunedinPACE*.

### 2.6 Statistical analysis

#### 2.6.1 Associations analysis

To assess the associations between epigenetic age metrics and phenotypes, we used two approaches to exclude the genetic confounding: (1) across twins analysis and (2) within MZ twin pairs analysis.

For continuous variables (FPG, HbA1c, and TyG index), we constructed mixed‐effects models with the R package *nlme*. In the first approach, across twins, we included twin pair ID as random intercepts to address the dependency within twin pairs. We used epigenetic age metrics as dependent variables and phenotypes as independent variables. A total of three hierarchical models were performed: (1) Model 1 adjusted for age, sex, education, and BMI; (2) Model 2 additionally adjusted for smoking pack‐years, alcohol consumption, and physical activity; and (3) Model 3 additionally adjusted for the use of hypoglycemic agents. In the second approach, within MZ twin pairs, we conducted the following formula: *E*(*Y*_*ij*_) = *β*_0_ + *β*_w_(*X*_*ij*_‐$\bar{X}$_*i*_) + *β*_b_ $\bar{X}$_*i*_ (Chen et al., 2020). The *Y*_*ij*_ is the epigenetic age metrics for twin *j* within the twin pair *i*, *X*_*ij*_ is the glycemic traits for twin *j* within the twin pair *i*, and $\bar{X}$_*i*_ is the mean of glycemic traits for the twin pair *i*. *β*_w_ is “within‐twin pair” regression coefficient, which represents the associations between epigenetic age metrics and glycemic traits after controlling for genetic confounding. For the categorical variable (T2DM), we performed doubly robust conditional generalized estimating equations (DRCGEE) to explore the associations, with the R package *drgee* (Isaksson et al., 2023; Pan & Bölte, 2020; Zetterqvist et al., 2016). First, across twins, the covariates are adjusted successively in Model 1, 2, and 3, while not adjusting for genetic confounders. Then, within MZ twin pairs, the twin pair ID was adjusted as the cluster.

Moreover, to explore the influence of sex, age, or education on associations of epigenetic age metrics with T2DM and glycemic traits, we conducted stratified analysis by sex (male, female), age (<50 years old and ≥50 years old), and education (low, middle, and high level) across twins.

For longitudinal data (*N* = 314), to investigate the associations between the longitudinal change rate of PCGrimAge/PCPhenoAge and phenotypes, we constructed mixed‐effect models treating the PCGrimAge rate or PCPhenoAge rate as the dependent variable and phenotypes of the baseline/follow‐up visit as the independent variable using similar aforementioned models.

#### 2.6.2 Cross‐lagged analysis

To understand the temporal relationships, we performed cross‐lagged analysis using structural equation modelling in participants who completed baseline and follow‐up visits (*N* = 314), with the R package *lavaan*. First, across twins, in the cross‐lagged models, we adjusted baseline age, sex, BMI, education, smoking pack‐years, alcohol consumption, physical activity, and the use of hypoglycemic agents as covariates. To control the correlations within twin pairs, we used the “cluster” option in the *lavaan* package. Then within MZ twin pairs, the difference in epigenetic age metrics within a pair were associated with the difference in glycemic traits within the same pair using the cross‐lagged models. Model fit was evaluated with the standard root mean square residual (SRMR) and comparative fit index (CFI). CFI > 0.95 and SRMR < 0.08 indicated an adequate fit (Hu & Bentler, 1999).

**FIGURE 1:**
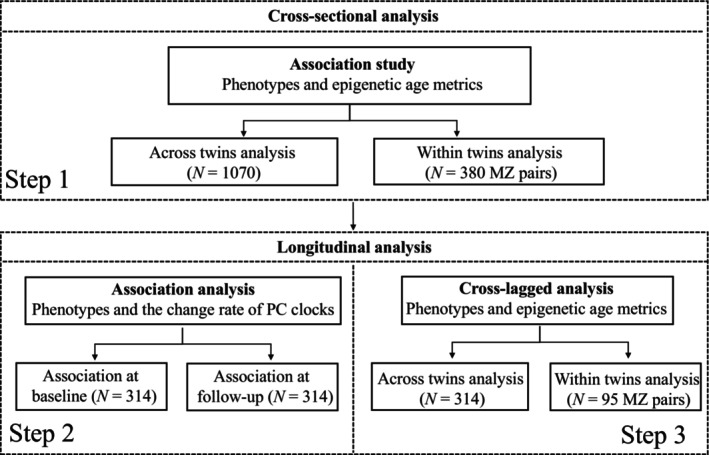
Analysis flowchart.

The analysis process is shown in Figure 1. Because of the non‐normal distribution, FPG and HbA1c were logarithmically transformed. To evaluate whether the associations we observed were independent of changes in blood cell composition, we conducted the sensitivity analysis by adjusting the blood cell compositions. The compositions were estimated using the *Houseman* method (Houseman et al., 2012). *p* Values were corrected with Bonferroni correction for multiple testing, and a corrected *p* < 0.05 was considered statistically significant. Statistical analysis was performed using R version 4.1.2.

## 3 RESULTS

### 3.1 Participant characteristics

Participant characteristics of all twins are presented in Table 1. Among the 1070 participants (535 twin pairs) included in the cross‐sectional analysis, the mean age was 49.9 years (age range: 19–82 years); 71.0% were monozygotic, and 68.0% were male. Of the 1070 twins, 106 (9.9%) were classified as T2DM. Among the 314 participants (157 twin pairs) who underwent both the baseline and follow‐up visits, 60.5% were monozygotic, and 60.8% were male; the baseline and follow‐up average ages were 50.4 (age range: 26–77 years) and 55.0 years (age range: 31–82 years), respectively. The average interval between the two visits was 4.6 years. Of twins included in both visits, the proportion with T2DM was 11.5% at the baseline visit and 16.9% at the follow‐up visit.

**TABLE 1:** The characteristics of study participants.

Variables	Overall samples (*N* = 1070)	Samples with both baseline and follow‐up visits (*N* = 314)	
		Baseline	Follow‐up
Demographics			
Chronological age, years	49.9 ± 12.2	50.4 ± 10.2	55.0 ± 10.1
Age group (%)			
<50 years old	512 (47.9)	152 (48.4)	88 (28.0)
≥50 years old	558 (52.1)	162 (51.6)	226 (72.0)
Male (%)	728 (68.0)	191 (60.8)	
Monozygotic (%)	760 (71.0)	190 (60.5)	
Education			
Low level	423 (39.5)	128 (40.8)	
Middle level	602 (56.3)	175 (55.7)	
High level	45 (4.2)	11 (3.5)	
Smoking status (%)			
Current smoker	347 (32.4)	98 (31.2)	91 (29.0)
Former smoker	138 (12.9)	26 (8.3)	35 (11.1)
Nonsmoker	585 (54.7)	190 (60.5)	188 (59.9)
Pack‐years of smoking (%)			
Nonsmoker	591 (55.2)	194 (61.8)	193 (61.5)
<30	295 (27.6)	75 (23.9)	64 (20.4)
≥30	162 (15.1)	42 (13.4)	56 (17.8)
Unknown	22 (2.1)	3 (0.9)	1 (0.3)
Drinking status (%)			
Current drinker	450 (42.2)	156 (50.2)	86 (27.6)
Former drinker	71 (6.6)	10 (3.2)	91 (29.3)
Nondrinker	546 (51.2)	145 (46.6)	134 (43.1)
Physical activity (%)			
High level	365 (34.1)	192 (61.1)	128 (40.7)
Middle level	186 (17.4)	79 (25.2)	69 (22.0)
Low level	519 (48.5)	43 (13.7)	117 (37.3)
BMI, kg/m^2^	24.8 ± 3.7	24.3 ± 3.5	24.3 ± 3.5
Measure of T2DM			
FPG, mmol/L	6.2 ± 2.5	6.0 ± 2.1	6.5 ± 2.8
HbA1c (%)	6.0 ± 1.3	6.0 ± 1.2	6.3 ± 1.5
TyG	8.81 ± 0.74	8.83 ± 0.72	8.91 ± 0.69
T2DM (%)	106 (9.9)	36 (11.5)	53 (16.9)
Hypoglycemic agents use (%)	113 (10.6)	32 (10.2)	48 (15.3)
Measure of epigenetic age metrics			
PCGrimAge, years	65.08 ± 10.00	65.10 ± 8.31	68.98 ± 8.20
PCPhenoAge, years	55.68 ± 10.47	58.48 ± 8.54	62.64 ± 8.16
PCGrimAA, years	0.00 ± 2.62	−0.15 ± 2.71	0.15 ± 2.71
PCPhenoAA, years	0.00 ± 4.39	−0.42 ± 4.56	0.41 ± 3.97
DunedinPACE	1.1 ± 0.1	1.1 ± 0.1	1.1 ± 0.1

*Note*: Data are presented as n (%) or mean ± standard deviation.

Abbreviations: BMI, body mass index; FPG, fasting plasma glucose; HbA1c, hemoglobin A1c; PCGrimAA, PCGrimAge acceleration; PCPhenoAA, PCPhenoAge acceleration; T2DM, Type 2 diabetes mellitus; TyG, triglyceride glucose index.

As expected, PCPhenoAge and PCGrimAge were highly correlated with chronological age (correlation coefficient > 0.90), and DunedinPACE showed a poor correlation with chronological age (correlation coefficient = 0.38). Three epigenetic age metrics (PCGrimAA, PCPhenoAA, and DunedinPACE) were positively correlated with each other (Figure S1).

Table S1 displays the intraclass twin correlations (ICC) for MZ and DZ twins for three epigenetic age metrics, three glycemic traits, and T2DM. All ICCs of MZ were greater than that of DZ for the cross‐sectional and longitudinal data, suggesting the genetic influences on these seven variables.

### 3.2 Cross‐sectional associations of epigenetic age metrics with T2DM and glycemic traits

First, across twins, after adjusting for multiple testing, at *p* < 0.0042, three epigenetic age metrics (PCGrimAA, PCPhenoAA, and DunedinPACE) were all associated with two glycemic traits (FPG and HbA1c), and DunedinPACE was also associated with TyG index in three models. After adjusting BMI, education, lifestyle factors, and the use of hypoglycemic agents (Model 3), each log (FPG, mmol/L) was associated with an increased 0.79 years of PCGrimAA (95% CI: 0.30, 1.27), an increased 1.82 years of PCPhenoAA (95% CI: 0.90, 2.74) or an increased 0.05 unit of DunedinPACE (95% CI: 0.03, 0.08). A unit increase in log (HbA1c, %) was associated with an increased 1.53 years of PCGrimAA (95% CI: 0.69, 2.37), an increased 2.79 years of PCPhenoAA (95% CI: 1.15, 4.43), or an increased 0.11 unit of DunedinPACE (95% CI: 0.07, 0.15). Besides, each unit of TyG index was associated with an increased 0.02 unit of DunedinPACE (95% CI: 0.01, 0.02) (Table S2).

Moreover, across twins, after adjusting for multiple testing, DunedinPACE was positively associated with three glycemic traits among twins aged 50 years and above, but not among those aged <50 years. Stratification by sex yielded similar results to the main results only among the men group. DunedinPACE was positively associated with FPG/HbA1c only in twins who had a low education level (Table S3).

Then, within MZ pairs, we only found the associations between DunedinPACE and three glycemic traits, and that between PCPhenoAA and FPG in Model 3 after adjusting for multiple testing (Table S4).

Table 2 demonstrates the result of the across‐twins analyses and within‐MZ‐pairs analyses in Model 3. In both analyses, we found positive associations between DunedinPACE and three glycemic traits, and positive associations between PCPhenoAA and FPG. This result indicates that these associations may not be influenced by genetic factors. Further adjustment for blood cell composition did not appreciably alter these associations (Table S5).

**TABLE 2:** Cross‐sectional associations between epigenetic age metrics and T2DM or glycemic trait.

Phenotypes	Epigenetic age metrics	Across twins^a^			Within MZ pairs^b^		
		*N* ^c^	Estimate (95% CI)	*p*	*N* ^d^	Estimate (95% CI)	*p*
FPG^e^	PCGrimAA	1051	**0.79 (0.30, 1.27)**	**0.001**	374	0.85 (0.05, 1.66)	0.038
	PCPhenoAA		**1.82 (0.90, 2.74)**	**<0.001**		**2.74 (1.28, 4.20)**	**<0.001**
	DunedinPACE		**0.05 (0.03, 0.08)**	**<0.001**		**0.07 (0.04, 0.11)**	**<0.001**
HbA1c^e^	PCGrimAA	1034	**1.53 (0.69, 2.37)**	**<0.001**	362	0.99 (−0.54, 2.51)	0.204
	PCPhenoAA		**2.79 (1.15, 4.43)**	**0.001**		2.58 (−0.20, 5.36)	0.069
	DunedinPACE		**0.11 (0.07, 0.15)**	**<0.001**		**0.11 (0.04, 0.18)**	**0.003**
TyG	PCGrimAA	1049	0.19 (0.00, 0.39)	0.048	372	0.10 (−0.25, 0.44)	0.588
	PCPhenoAA		0.16 (−0.21, 0.53)	0.400		0.05 (−0.59, 0.69)	0.877
	DunedinPACE		**0.02 (0.01, 0.03)**	**<0.001**		**0.02 (0.01, 0.04)**	**0.004**
T2DM	PCGrimAA	1004	0.05 (−0.53, 0.64)	0.855	363	−0.18 (−0.81, 0.44)	0.565
	PCPhenoAA		1.17 (0.18, 2.17)	0.021		0.48 (−0.40, 1.36)	0.287
	DunedinPACE		0.02 (0.00, 0.05)	0.069		0.02 (−0.01, 0.05)	0.237

*Note*: Significant results with *p <* 0.05/(3 × 4) = 0.0042 were highlighted in bold font (3: Three measures of epigenetic age metrics; 4: Four phenotypes).

Abbreviation: CI, confidence interval; FPG, fasting plasma glucose; HbA1c, hemoglobin A1c; MZ, monozygotic twin; PCGrimAA, PCGrimAge acceleration; PCPhenoAA, PCPhenoAge acceleration; T2DM, Type 2 diabetes mellitus; TyG, triglyceride glucose index.

^a^ We adjusted for age, sex, education, BMI, smoking pack‐years, alcohol consumption, physical activity, and the use of hypoglycemic agents. The estimate represented the change in the phenotypes associated with a 1 year or 1 increase in the epigenetic age metrics.

^b^ We adjusted for education, BMI, smoking pack‐years, alcohol consumption, physical activity, and the use of hypoglycemic agents. The estimate represents the association coefficient after controlling for genetic confounding.

^c^ Number of twins.

^d^ Number of MZ twin pairs.

^e^ Natural log transformation was performed due to the skewed distribution of the variable.

### 3.3 Associations between the longitudinal change rate of epigenetic age and T2DM and glycemic traits

The longitudinal change rate of PCGrimAge between baseline and follow‐up visits was 0.84 (ranged from −0.12 to 2.43), and that of PCPhenoAge was 0.93 (ranged from −1.35 to 5.49).

At *p* < 0.05, TyG index at baseline was positively associated with PCGrimAge rate and PCPhenoAge rate. However, after multiple testing, only the positive association between TyG index at baseline and PCPhenoAge rate were still statistically significant (Table S6).

### 3.4 Cross‐lagged analysis for epigenetic age metrics and T2DM and glycemic traits

**FIGURE 2:**
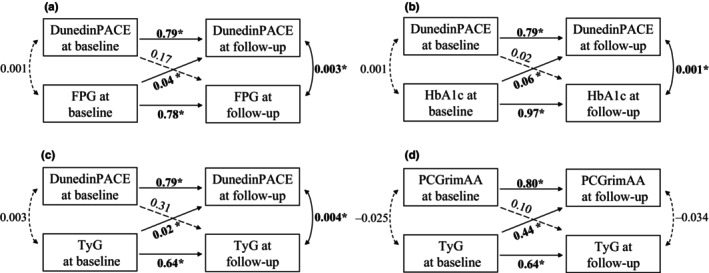
Cross‐lagged relationships between epigenetic age metrics and glycemic traits across twins. FPG, fasting plasma glucose; HbA1c, hemoglobin A1c; TyG, triglyceride glucose index; *: Significant results with *p <* 0.05/(3 × 4) = 0.0042 were highlighted in bold font (3: Three measures of epigenetic age metrics; 4: Four phenotypes).

First, across twins, we utilized baseline and follow‐up data to construct cross‐lagged panel models and the goodness of fit measures for these models showed that all the models perform well (Table S7). We only found significant positive cross‐lagged associations from three glycemic traits to DunedinPACE and that from TyG index to PCGrimAA at the *p* < 0.0042 level (Figure 2). The autoregressive paths were all significant, indicating the stability of DunedinPACE, PCGrimAA, and three glycemic traits over time. As the model predicted, individuals with higher levels of TyG index would have an increase in PCGrimAA after 4.6 years; and individuals with higher levels of glycemic traits (FPG, HbA1c, and TyG index) would have an increase in DunedinPACE after 4.6 years.

Then within MZ pairs, all models performed well. However, the temporal relationships were insignificant after multiple testing (Table S8).

## 4 DISCUSSION

In this study, positive associations were identified between DunedinPACE and three glycemic traits; and between PCPhenoAA and FPG. These associations were found in both across‐twins and within‐MZ‐pairs analyses, showing that they were “pure” and not confounded by genetic factors. We also found that three glycemic traits precede DunedinPACE increases, and TyG index precedes PCGrimAA increases. Taken together, glycemic traits were positively associated with epigenetic age metrics, especially DunedinPACE; and glycemic traits could be involved in the progress of epigenetic aging.

Our cross‐sectional results contribute to the growing evidence for the associations between epigenetic age metrics and glycemic traits. Across twins, we found positive associations between PC clocks (PCGrimAA and PCPhenoAA) and FPG, and that between PC clocks and HbA1c, consistent with previous findings using original clocks (GrimAA, PhenoAA) (Ammous et al., 2021; Arpon et al., 2019; Lee & Park, 2020; Lo & Lin, 2022; Lundgren et al., 2022; Stevenson et al., 2019). In addition, positive associations between DunedinPACE and all three glycemic traits in both analyses are robust. Only one study based on 2474 Chinese Taiwan participants found that DunedinPACE, rather than other epigenetic age metrics, was associated with more health outcomes (Lin, 2023). This conclusion is consistent with our findings.

In stratified analyses, it appeared that the positive associations between epigenetic age metrics and glycemic traits were only in males, or with a low educational level. Plausible explanations are the small sample size of the female group and the high level of education group (Table 1), or the possibility of shorter lifespans in males (Oblak et al., 2021; Phyo et al., 2023) and low health literacy in people with the low level of education (Graf et al., 2024; Oblak et al., 2021). The associations between glycemic traits and DunedinPACE may be more common in the elder group (aged 50 years and above) due to the abnormal glucose metabolism and insulin resistance associated with aging (Li et al., 2021; Palmer, Xu, et al., 2019; Safwan‐Zaiter et al., 2022).

We also explored the temporal mechanism between epigenetic age metrics and glycemic traits. PCGrimAge rate and PCPhenoAge rate indicated the pace of aging, which models the longitudinal changing speed of epigenetic clocks compared to the passage of chronological age. The positive associations between baseline TyG index and PCPhenoAge rate indicated the potential influence of TyG index on the pace of aging. Only a longitudinal study focused on the associations between the change rate of epigenetic age and T2DM, and showed the potential of the GrimAge rate to predict T2DM (Kim et al., 2021). Future studies are warranted to understand further the mechanisms and utility of the change rate of PCGrimAge and PCPhenoAge.

The cross‐lagged analysis showed that glycemic traits precede increases in DunedinPACE and PCGrimAA. Elevated FPG and HbA1c cause inflammation and then damage endothelial cells through multiple signaling pathways, thus causing long‐term oxidative stress (Cruz et al., 2013; Piao et al., 2014). TyG is an insulin resistance index, and insulin resistance is also linked to inflammation and oxidative stress, which are part of the mechanism of aging (Bondia‐Pons et al., 2012; Garm et al., 2013). The temporal relationships linking glycemic traits to DunedinPACE and PCGrimAA may reflect the influence of glucose metabolism and insulin resistance on epigenetic aging. Evidence from previous studies yielded temporal relationships linking glycemic traits to CpG sites (Hong et al., 2022; Vigorelli et al., 2019). The effect of glycemic traits on methylation was confirmed using DunedinPACE and PCGrimAA, the summary measures of methylation. No significant temporal relationships were found in within‐MZ‐pairs analyses; this is probably due to the relatively small sample size (95 pairs). Larger twin studies are needed to explore whether shared genetic factors influence the temporal relationship.

Our study only found the positive associations of T2DM with DunedinPACE and PCPhenoAA in cross‐sectional analysis, and the associations were statistically insignificant after adjusting lifestyle factors. Multiple studies have shown the associations of PhenoAA/DunedinPACE with T2DM (Lin, 2023; Lu et al., 2019). A Mendelian randomization study found that T2DM was the risk factor and the cause of GrimAA (Kong et al., 2023). We also found the temporal relationship of T2DM at baseline to GrimAA at follow‐up in the cross‐lagged analysis, but it was no longer statistically significant after multiple testing. The smaller T2DM case size in this study, 106 in cross‐sectional analysis and 36 in longitudinal analysis, might explain why few associations between T2DM and epigenetic age metrics were statistically significant in this study.

Different epigenetic age metrics represent various aspects of aging (Liu et al., 2020). The PCGrimAA and PCPhenoAA capture the degree of aging, while DunedinPACE estimates how fast aging processes are occurring at a single time. They may be complementary (Moqri et al., 2023). The evidence presented in this study suggests that compared with PCGrimAA and PCPhenoAA, DunedinPACE had more associations with glycemic traits. Moreover, the longitudinal relationship of glycemic traits to DunedinPACE could partly suggest that DunedinPACE is more sensitive to changes of glucose metabolism and insulin resistance in aging processes. In our sensitivity analysis, the associations were not attenuated after adjusting for blood cell composition. This suggests that the observed associations are not caused by age‐related changes in blood cell composition, and that all epigenetic age metrics (PCGrimAA, PCPhenoAA, and DunedinPACE) capture the cell intrinsic characteristics associated with aging (Ammous et al., 2021).

Our study has several strengths. First, we applied these three epigenetic clocks, especially PC clocks, which were more reliable than original clocks, for the first time in Chinese twin populations. Although they developed based on European ancestries, their correlation with chronological age is close to previous studies (Belsky et al., 2022; Levine et al., 2018; Lu et al., 2019), indicating that epigenetic clocks are also applicable to Chinese populations. Then, we used co‐twin control analysis to identify whether shared genetic factors confounded the associations observed. Additionally, we tested the longitudinal relationship between epigenetic age metrics and T2DM or glycemic traits, which provide clues to their causal relationship. However, our study was also subject to potential limitations. The subjects in this study were from CNTR, a national register of twins recruited as volunteers, they were not representative of the Chinese population. The low number of T2DM cases and MZ twin pairs of longitudinal research may result in insufficient statistical power of the study. We also did not collect information on T2DM onset. Future studies with a larger sample size should investigate the temporal relationship between epigenetic aging and the trajectories of the change in diabetes status.

## 5 CONCLUSION

In conclusion, we considered dual evaluation of aging and applied three epigenetic age metrics in this study to identify the associations between epigenetic aging and T2DM or glycemic traits. Hence, we conducted a co‐twin control study and found that these associations were not due to shared genetic factors. We also found that glycemic traits precede increases in DunedinPACE or PCGrimAA. Lowering the levels of glycemic traits may reduce DunedinPACE and PCGrimAA levels, thereby mitigating age‐related comorbidities. More experimental studies are needed to confirm this in the future.

## AUTHOR CONTRIBUTIONS

Wenjing Gao: Conceptualization, methodology, and writing—review and editing; Zengchang Pang, Min Yu, Hua Wang, Xianping Wu, and Yu Liu: Investigation; Xuanming Hong, Weihua Cao, Jun Lv, Canqing Yu, Tao Huang, Dianjianyi Sun, Chunxiao Liao, Yuanjie Pang, and Runhua Hu: Writing—review and editing; Liming Li: Supervision, project administration, funding acquisition; Ke Miao: software, and writing—original draft.

## FUNDING INFORMATION

This study was funded by the National Natural Science Foundation of China (82073633, 82373659, and 81573223), the Special Fund for Health Scientific Research in the Public Welfare (201502006 and 201002007), and the Peking University Outstanding Discipline Construction Project of Epidemiology and Biostatistics.

## CONFLICT OF INTEREST STATEMENT

The authors declare that they have no known competing financial interests or personal relationships that could have appeared to influence the work reported in this article.

## Supporting information


Appendix S1

## Data Availability

The data that support the findings of this study are available from the corresponding author upon reasonable request.
