# Supplementary material for: Association between epigenetic age and type 2 diabetes mellitus or glycemic traits: A longitudinal twin study
Source: Aging Cell. 2024 Apr 25;23(7):e14175. doi: 10.1111/acel.14175 (PMC11258448; doi:10.1111/acel.14175)
Supplement: Supplementary file 1 — Appendix S1 [file ACEL-23-e14175-s001.docx]

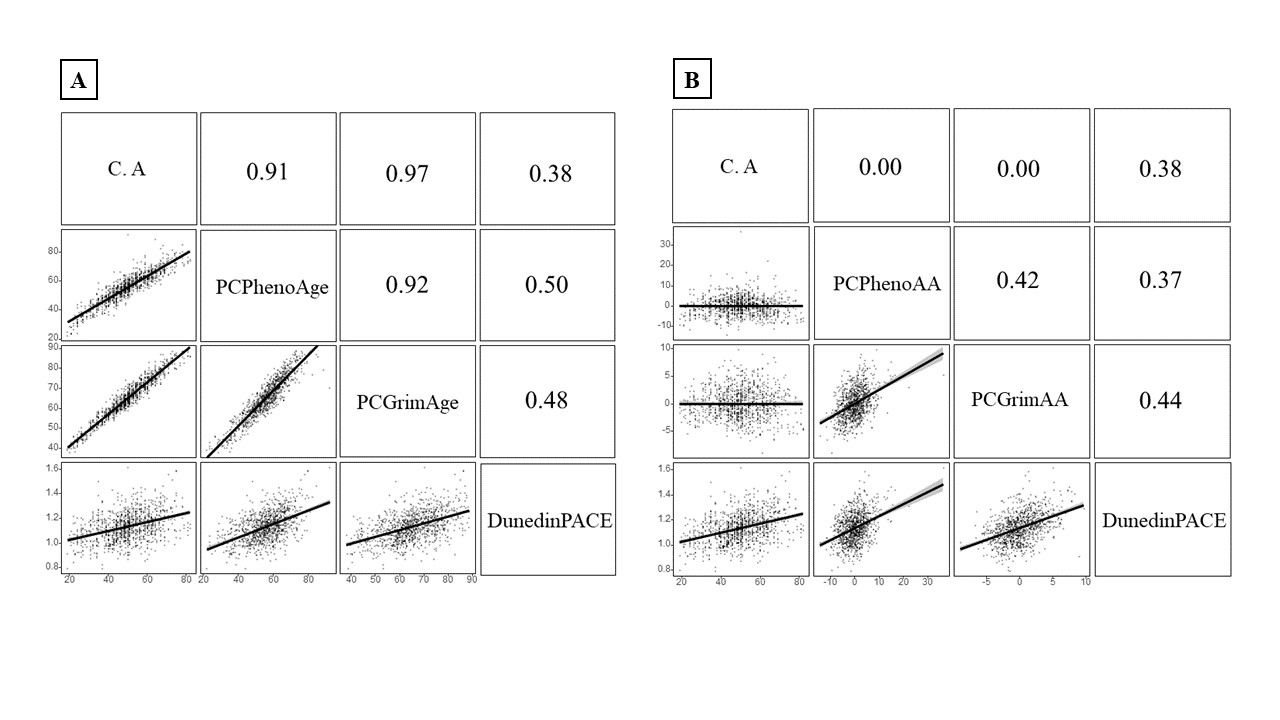


**Supplemental** **Figure** 1. Pairwise scatter plots of epigenetic age metrics and chronological age

1. Pairwise scatter plots showing the Pearson correlation coefficient between chronological age and epigenetic clocks.
2. Pairwise scatter plots showing the Pearson correlation coefficient between epigenetic age acceleration and epigenetic clocks.

The number in the top right corner represents the Pearson correlation coefficient.

Abbreviations: C. A, chronological age; PCGrimAA, PCGrimAge acceleration; PCPhenoAA, PhenoAge acceleration

**Supplemental Table 1.** The intraclass correlations of epigenetic age metrics, glycemic traits and T2DM

| Phenotypes | Cross-sectional ICC  (95% *CI*) | | ICC at baseline  (95% *CI*) | | ICC at follow-up  (95% *CI*) | |
| --- | --- | --- | --- | --- | --- | --- |
|  | MZ pairs  (N=380) | DZ pairs  (N=155) | MZ pairs  (N=95) | DZ pairs  (N=62) | MZ pairs  (N=95) | DZ pairs  (N=62) |
| FPG | 0.59  (0.52, 0.65) | 0.08  (-0.08, 0.24) | 0.63  (0.49, 0.74) | 0.26  (0.01, 0.48) | 0.68  (0.55, 0.78) | 0.04  (-0.22, 0.29) |
| HbA1c | 0.67  (0.61, 0.73) | 0.21  (0.05, 0.35) | 0.62  (0.48, 0.73) | 0.19  (-0.06, 0.42) | 0.73  (0.62, 0.82) | 0.05  (-0.21, 0.29) |
| TyG | 0.63  (0.56, 0.69) | 0.12  (-0.04, 0.27) | 0.67  (0.54, 0.77) | 0.04  (-0.21, 0.28) | 0.61  (0.46, 0.72) | -0.10  (-0.35, 0.15) |
| T2DM | 0.37  (0.28, 0.45) | -0.07  (-0.22, 0.09) | 0.43  (0.21, 0.62) | 0.38  (0.19, 0.54) | 0.49  (0.32, 0.63) | 0.35  (0.11, 0.55) |
| PCGrimAA | 0.68  (0.63, 0.73) | 0.31  (0.16, 0.45) | 0.76  (0.66, 0.83) | 0.36  (0.13, 0.56) | 0.71  (0.59, 0.79) | 0.21  (-0.04, 0.44) |
| PCPhenoAA | 0.61  (0.54, 0.67) | 0.47  (0.34, 0.58) | 0.78  (0.69, 0.85) | 0.56  (0.37, 0.71) | 0.61  (0.47, 0.72) | 0.22  (-0.03, 0.45) |
| DunedinPACE | 0.65  (0.58, 0.70) | 0.43  (0.29, 0.55) | 0.66  (0.53, 0.76) | 0.36  (0.12, 0.56) | 0.54  (0.38, 0.67) | 0.38  (0.15, 0.58) |

Abbreviations: ICC, intraclass correlations; FPG, fasting plasma glucose; HbA1c, hemoglobin A1c; TyG, triglyceride glucose index; T2DM, Type 2 diabetes mellitus; PCGrimAA, PCGrimAge acceleration; PCPhenoAA, PCPhenoAge acceleration.

**Supplemental Table 2.** Cross-sectional association of epigenetic age metrics with phenotypes across twins

| Phenotypes | Epigenetic age metrics | Model 1 | | Model 2 | | Model 3 | |
| --- | --- | --- | --- | --- | --- | --- | --- |
|  |  | Estimate  (95%*CI*) | *P* | Estimate  (95%*CI*) | *P* | Estimate  (95%*CI*) | *P* |
| FPG  (N=1051) | PCGrimAA | **0.78**  **(0.32, 1.24)** | **0.001** | **0.75**  **(0.3, 1.19)** | **0.001** | **0.79**  **(0.30, 1.27)** | **0.001** |
|  | PCPhenoAA | **1.84**  **(0.98, 2.70)** | **<0.001** | **1.74**  **(0.88, 2.6)** | **<0.001** | **1.82**  **(0.90, 2.74)** | **<0.001** |
|  | DunedinPACE | **0.06**  **(0.03, 0.08)** | **<0.001** | **0.05**  **(0.03, 0.07)** | **<0.001** | **0.05**  **(0.03, 0.08)** | **<0.001** |
| HbA1c  (N=1034) | PCGrimAA | **1.65**  **(0.85, 2.44)** | **<0.001** | **1.41**  **(0.65, 2.17)** | **<0.001** | **1.53**  **(0.69, 2.37)** | **<0.001** |
|  | PCPhenoAA | **2.77**  **(1.26, 4.27)** | **<0.001** | **2.71**  **(1.21, 4.21)** | **<0.001** | **2.79**  **(1.15, 4.43)** | **0.001** |
|  | DunedinPACE | **0.11**  **(0.07, 0.14)** | **<0.001** | **0.10**  **(0.06, 0.14)** | **<0.001** | **0.11**  **(0.07, 0.15)** | **<0.001** |
| TyG  (N=1049) | PCGrimAA | 0.28  (0.08, 0.47) | 0.005 | 0.20  (0.01, 0.39) | 0.035 | 0.19  (0.00, 0.39) | 0.048 |
|  | PCPhenoAA | 0.25  (-0.12, 0.62) | 0.182 | 0.19  (-0.18, 0.55) | 0.322 | 0.16  (-0.21, 0.53) | 0.400 |
|  | DunedinPACE | **0.02**  **(0.01, 0.03)** | **<0.001** | **0.02**  **(0.01, 0.03)** | **<0.001** | **0.02**  **(0.01, 0.03)** | **<0.001** |
| T2DM  (N=1004) | PCGrimAA | 0.25  (-0.17, 0.67) | 0.241 | 0.31  (-0.12, 0.73) | 0.155 | 0.05  (-0.53, 0.64) | 0.855 |
|  | PCPhenoAA | **1.30**  **(0.54, 2.06)** | **0.001** | 1.11  (0.33, 1.89) | 0.005 | 1.17  (0.18, 2.17) | 0.021 |
|  | DunedinPACE | **0.04**  **(0.01, 0.06)** | **0.001** | **0.03**  **(0.01, 0.06)** | **0.002** | 0.02  (0.00, 0.05) | 0.069 |

Abbreviation: CI, confidence interval; FPG, fasting plasma glucose; HbA1c, hemoglobin A1c; TyG, triglyceride glucose index; T2DM, Type 2 diabetes mellitus; PCGrimAA, PCGrimAge acceleration; PCPhenoAA, PCPhenoAge acceleration.

Model 1: adjusted for age, sex, education and BMI;

Model 2: Model 1 +smoking pack-years, alcohol consumption and physical activity;

Model 3: Model 2 + the use of hypoglycemic agents.

Significant results with *P* < 0.05/(3 x 4) = 0.0042 are highlighted in bold font. (3: Three measures of epigenetic age metrics; 4: Four phenotypes)

The estimate represented the change in the epigenetic age metrics associated with one-unit increase in the phenotypes;

**Supplemental Table 3.** Cross-sectional association of epigenetic age metrics with phenotypes across twins in stratified analyses

| Phenotype | Subgroups | PCGrimAA | | PCPhenoAA | | DunedinPACE | |
| --- | --- | --- | --- | --- | --- | --- | --- |
|  |  | β(95%CI) | *P* | β(95%CI) | *P* | β(95%CI) | *P* |
| **Stratification by age^a^** | | | | | | | |
| FPG | <50 years old | 0.45 (-0.34, 1.24) | 0.261 | 0.87 (-0.56, 2.30) | 0.233 | 0.02 (-0.02, 0.06) | 0.268 |
|  | ≥50 years old | 0.87 (0.24, 1.51) | 0.007 | 1.87 (0.63, 3.10) | 0.003 | **0.08 (0.04, 0.11)** | **<0.001** |
| HBA1C | <50 years old | 1.52 (0.21, 2.84) | 0.024 | 3.31 (0.91, 5.72) | 0.007 | 0.09 (0.03, 0.15) | 0.005 |
|  | ≥50 years old | 1.46 (0.33, 2.59) | 0.012 | 2.03 (-0.21, 4.27) | 0.076 | **0.13 (0.08, 0.18)** | **<0.001** |
| TyG | <50 years old | 0.20 (-0.06, 0.45) | 0.138 | -0.09 (-0.59, 0.41) | 0.722 | 0.02 (0.00, 0.03) | 0.016 |
|  | ≥50 years old | 0.18 (-0.11, 0.47) | 0.230 | 0.21 (-0.36, 0.77) | 0.472 | **0.03 (0.01, 0.04)** | **<0.001** |
| T2DM | <50 years old | -0.44 (-1.19, 0.31) | 0.252 | 0.53 (-0.82, 1.87) | 0.444 | 0.02 (-0.01, 0.04) | 0.236 |
|  | ≥50 years old | 0.07 (-0.70, 0.83) | 0.865 | 1.00 (-0.31, 2.32) | 0.133 | 0.03 (0.00, 0.06) | 0.089 |
| **Stratification by gender^a^** | | | | | | | |
| FPG | male | **1.04 (0.48, 1.61)** | **<0.001** | **1.90 (0.94, 2.86)** | **<0.001** | **0.05 (0.03, 0.08)** | **<0.001** |
|  | female | 0.06 (-0.91, 1.03) | 0.902 | 1.61 (-0.73, 3.96) | 0.175 | **0.08 (0.03, 0.13)** | **0.001** |
| HBA1C | male | **1.73 (0.70, 2.76)** | **0.001** | **2.86 (1.03, 4.69)** | **0.002** | **0.10 (0.05, 0.15)** | **<0.001** |
|  | female | 1.13 (-0.41, 2.67) | 0.149 | 2.93 (-0.81, 6.67) | 0.123 | **0.14 (0.06, 0.21)** | **<0.001** |
| TyG | male | 0.31 (0.08, 0.54) | 0.008 | 0.32 (-0.08, 0.72) | 0.119 | **0.02 (0.01, 0.03)** | **<0.001** |
|  | female | 0.01 (-0.35, 0.37) | 0.972 | 0.01 (-0.87, 0.88) | 0.989 | 0.02 (0.01, 0.04) | 0.009 |
| T2DM | male | 0.16 (-0.47, 0.79) | 0.623 | 1.13 (-0.01, 2.26) | 0.052 | 0.01 (-0.01, 0.04) | 0.310 |
|  | female | -0.48 (-1.58, 0.61) | 0.390 | 0.94 (-0.88, 2.76) | 0.313 | 0.03 (-0.01, 0.08) | 0.142 |
| **Stratification by education^b^** | | | | | | | |
| FPG | low | 0.88 (0.22, 1.54) | 0.009 | 1.90 (0.65, 3.15) | 0.003 | **0.07 (0.03, 0.10)** | **<0.001** |
|  | middle | 0.70 (-0.02, 1.42) | 0.057 | 1.72 (0.28, 3.16) | 0.019 | 0.04 (0.01, 0.07) | 0.014 |
|  | high | 0.42 (-3.32, 4.16) | 0.745 | 0.48 (-4.60, 5.56) | 0.783 | 0.04 (-0.16, 0.23) | 0.596 |
| HbA1c | low | 1.33 (0.13, 2.54) | 0.030 | 2.55 (0.26, 4.84) | 0.029 | **0.14 (0.08, 0.20)** | **<0.001** |
|  | middle | 1.68 (0.48, 2.89) | 0.006 | 3.34 (0.94, 5.73) | 0.006 | 0.08 (0.02, 0.13) | 0.008 |
|  | high | 1.38 (-7.72, 10.48) | 0.581 | 4.46 (-8.95, 17.87) | 0.289 | 0.21 (-0.27, 0.68) | 0.199 |
| TyG | low | 0.24 (-0.06, 0.53) | 0.113 | 0.48 (-0.08, 1.05) | 0.093 | 0.02 (0.01, 0.04) | 0.005 |
|  | middle | 0.16 (-0.10, 0.43) | 0.220 | -0.03 (-0.56, 0.50) | 0.912 | 0.02 (0.01, 0.03) | 0.004 |
|  | high | 0.36 (-1.13, 1.85) | 0.500 | 0.39 (-1.83, 2.61) | 0.614 | 0.03 (-0.05, 0.11) | 0.380 |
| T2DM | low | -0.01 (-0.83, 0.81) | 0.984 | 1.84 (0.04, 3.64) | 0.045 | 0.05 (0.01, 0.09) | 0.029 |
|  | middle | -0.10 (-0.92, 0.71) | 0.802 | 0.40 (-0.74, 1.54) | 0.490 | <0.01 (-0.03, 0.03) | 0.814 |
|  | high | -0.41 (-2.89, 2.07) | 0.743 | -2.47 (-5.05, 0.11) | 0.061 | -0.12 (-0.19, -0.04) | 0.004 |

Abbreviation: CI, confidence interval; FPG, fasting plasma glucose; HbA1c, hemoglobin A1c; TyG, triglyceride glucose index; T2DM, Type 2 diabetes mellitus; PCGrimAA, PCGrimAge acceleration; PCPhenoAA, PCPhenoAge acceleration.

We adjusted for age, sex, BMI, education, smoke pack, alcohol consumption, physical activity, and the use of hypoglycemic agents;

a: Significant results with *P <* 0.05 / (3 x 4 x 2) = 0.0021 were highlighted in bold font. (3: Three epigenetic age metrics; 4: Four phenotypes; 2: the number of subgroups for gender or age)

b: Significant results with *P <* 0.05 / (3 x 4 x 3) = 0.0013 were highlighted in bold font. (3: Three epigenetic age metrics; 4: Four phenotypes; 3: the number of subgroups for education or smoke pack)

The estimate represented the change in the epigenetic age metrics associated with one-unit increase in the phenotypes;

**Supplemental Table 4.** Cross-sectional association of epigenetic age metrics with phenotypes using within-twin effect models in MZ twin pairs

| Phenotype | Epigenetic age metrics | Model 1 | | Model 2 | | Model 3 | | |
| --- | --- | --- | --- | --- | --- | --- | --- | --- |
|  |  | Estimate  (*95% CI*) | *P* | Estimate  (*95% CI*) | *P* | Estimate  (*95% CI*) | *P* |  |
| FPG  (N=374 pairs) | GrimAA | 0.82  (0.04, 1.61) | 0.039 | 0.83  (0.03, 1.63) | 0.043 | 0.85  (0.05, 1.66) | 0.038 |  |
|  | PhenoAA | **2.65**  **(1.20, 4.1)** | **<0.001** | **2.73**  **(1.27, 4.19)** | **<0.001** | **2.74**  **(1.28, 4.20)** | **<0.001** |  |
|  | DunedinPACE | **0.07**  **(0.03, 0.10)** | **<0.001** | **0.07**  **(0.04, 0.11)** | **<0.001** | **0.07**  **(0.04, 0.11)** | **<0.001** |  |
| HbA1c  (N=362 pairs) | GrimAA | 1.33  (-0.13, 2.79) | 0.073 | 0.86  (-0.64, 2.36) | 0.259 | 0.99  (-0.54, 2.51) | 0.204 |  |
|  | PhenoAA | 2.33  (-0.37, 5.04) | 0.091 | 2.52  (-0.21, 5.25) | 0.071 | 2.58  (-0.20, 5.36) | 0.069 |  |
|  | DunedinPACE | **0.10**  **(0.03, 0.17)** | **0.003** | **0.11**  **(0.04, 0.17)** | **0.003** | **0.11**  **(0.04, 0.18)** | **0.003** |  |
| TyG  (N=372 pairs) | GrimAA | 0.14  (-0.20, 0.48) | 0.434 | 0.10  (-0.25, 0.44) | 0.582 | 0.10  (-0.25, 0.44) | 0.588 |  |
|  | PhenoAA | 0.06  (-0.58, 0.70) | 0.855 | 0.04  (-0.59, 0.68) | 0.894 | 0.05  (-0.59, 0.69) | 0.877 |  |
|  | DunedinPACE | **0.02**  **(0.01, 0.04)** | **0.004** | 0.02  (0.01, 0.04) | 0.005 | **0.02**  **(0.01, 0.04)** | **0.004** |  |
| T2DM  (N=363 pairs) | GrimAA | -0.30  (-0.84, 0.24) | 0.271 | -0.27  (-0.76, 0.21) | 0.268 | -0.18  (-0.81, 0.44) | 0.565 |  |
|  | PhenoAA | 0.42  (-0.30, 1.14) | 0.250 | 0.43  (-0.29, 1.15) | 0.244 | 0.48  (-0.40, 1.36) | 0.287 |  |
|  | DunedinPACE | 0.01  (-0.01, 0.04) | 0.396 | 0.01  (-0.01, 0.04) | 0.412 | 0.02  (-0.01, 0.05) | 0.237 |  |

Abbreviation: CI, confidence interval; FPG, fasting plasma glucose; HbA1c, hemoglobin A1c; TyG, triglyceride glucose index; T2DM, Type 2 diabetes mellitus; PCGrimAA, PCGrimAge acceleration; PCPhenoAA, PCPhenoAge acceleration.

Model 1: adjusted for BMI and education;

Model 2: Model 1 + smoking pack-years, alcohol consumption and physical activity;

Model 3: Model 2 + the use of hypoglycemic agents.

Significant results with *P <* 0.05 / (3 x 4) = 0.0042 were highlighted in bold font. (3: Three measures of epigenetic age metrics; 4: Four phenotypes)

**Supplemental Table 5.** Cross-sectional association of epigenetic age metrics with phenotypes after adjusting blood cell compositions.

| Phenotypes | Epigenetic age metrics | Across twins^a^ | | | Within MZ pairs^b^ | | |
| --- | --- | --- | --- | --- | --- | --- | --- |
|  |  | N^c^ | Estimate  (95%*CI*) | *P* | N^d^ | Estimate  (95%*CI*) | *P* |
| FPG | PCGrimAA |  | 0.56  (0.14, 0.97) | 0.008 | 374 | 0.85  (0.05, 1.66) | 0.038 |
|  | PCPhenoAA | 1051 | **1.42**  **(0.63, 2.2)** | **<0.001** |  | **2.74**  **(1.28, 4.20)** | **<0.001** |
|  | DunedinPACE |  | **0.05**  **(0.03, 0.07)** | **<0.001** |  | **0.07**  **(0.04, 0.11)** | **<0.001** |
| HbA1c | PCGrimAA |  | **1.33**  **(0.61, 2.05)** | **<0.001** | 362 | 1.02  (-0.12, 2.17) | 0.080 |
|  | PCPhenoAA | 1034 | **2.72**  **(1.29, 4.14)** | **<0.001** |  | 2.61  (0.41, 4.82) | 0.020 |
|  | DunedinPACE |  | **0.10**  **(0.07, 0.14)** | **<0.001** |  | **0.10**  **(0.04, 0.17)** | **0.002** |
| TyG | PCGrimAA |  | 0.18  (0.01, 0.35) | 0.033 | 372 | 0.10  (-0.25, 0.44) | 0.588 |
|  | PCPhenoAA | 1049 | 0.35  (0.03, 0.67) | 0.034 |  | 0.05  (-0.59, 0.69) | 0.877 |
|  | DunedinPACE |  | **0.02**  **(0.01, 0.03)** | **<0.001** |  | **0.02**  **(0.01, 0.04)** | **0.004** |
| T2DM | PCGrimAA |  | -0.11  (-0.64, 0.41) | 0.673 | 363 | -0.18  (-0.81, 0.44) | 0.565 |
|  | PCPhenoAA | 1004 | 1.08  (0.20, 1.96) | 0.017 |  | 0.48  (-0.40, 1.36) | 0.287 |
|  | DunedinPACE |  | 0.02  (-0.01, 0.04) | 0.191 |  | 0.02  (-0.01, 0.05) | 0.237 |

Abbreviation: CI, confidence interval; FPG, fasting plasma glucose; HbA1c, hemoglobin A1c; TyG, triglyceride glucose index; T2DM, Type 2 diabetes mellitus; PCGrimAA, PCGrimAge acceleration; PCPhenoAA, PCPhenoAge acceleration; MZ, monozygotic twin;

a: We adjusted for age, sex, BMI, education, smoke pack, alcohol consumption, physical activity, the use of hypoglycemic agents and blood cell compositions;

b: We adjusted for BMI, education, smoke pack, alcohol consumption, physical activity, the use of hypoglycemic agents and blood cell compositions;

c: the number of twins;

d: the number of MZ twin pairs;

Significant results with P < 0.05 / (3 x 4) = 0.0042 were highlighted in bold font. (3: Three measures of epigenetic age metrics; 4: Four phenotypes)

**Supplemental Table 6.** Association of the longitudinal change rate of epigenetic age with T2DM or glycemic traits at baseline or follow-up

| Phenotypes | Epigenetic age metrics | Baseline | | Follow-up | |
| --- | --- | --- | --- | --- | --- |
|  |  | Estimate  (95%*CI*) | *P* | Estimate  (95%*CI*) | *P* |
| FPG  (N=307) | PCGrimAge rate | 0.13  (-0.07, 0.32) | 0.197 | 0.03  (-0.14, 0.19) | 0.726 |
|  | PCPhenoAge rate | 0.28  (-0.12, 0.68) | 0.170 | 0.22  (-0.12, 0.56) | 0.198 |
| HbA1c  (N=312) | PCGrimAge rate | 0.14  (-0.17, 0.44) | 0.374 | 0.02  (-0.24, 0.27) | 0.894 |
|  | PCPhenoAge rate | 0.44  (-0.19, 1.07) | 0.166 | 0.27  (-0.25, 0.8) | 0.309 |
| TyG  (N=307) | PCGrimAge rate | 0.09  (0.02, 0.16) | 0.009 | 0.02  (-0.05, 0.09) | 0.520 |
|  | PCPhenoAge rate | **0.22**  **(0.07, 0.37)** | **0.004** | 0.01  (-0.13, 0.16) | 0.848 |
| T2DM  (N=306) | PCGrimAge rate | 0.09  (-0.16, 0.34) | 0.488 | 0.03  (-0.12, 0.18) | 0.681 |
|  | PCPhenoAge rate | -0.29  (-0.99, 0.4) | 0.411 | -0.05  (-0.38, 0.28) | 0.760 |

Abbreviation: CI, confidence interval; FPG, fasting plasma glucose; HbA1c, hemoglobin A1c; TyG, triglyceride glucose index; T2DM, Type 2 diabetes mellitus; PCGrimAA, PCGrimAge acceleration; PCPhenoAA, PCPhenoAge acceleration

We adjusted for age, sex, BMI, education, smoking pack-years, alcohol consumption, physical activity and the use of hypoglycemic agents at baseline;

Significant results with P < 0.05 / (2 x 4) = 0.0063 were highlighted in bold font. (2: Two measures of longitudinal change rate of epigenetic age; 4: Four phenotypes)

**Supplemental Table 7.** Cross-lagged relationships between epigenetic age metrics and T2DM or glycemic traits across twins

| Phenotypes | N | epigenetic age metrics _base_→ phenotypes _follow_ | | phenotypes _base_→ epigenetic age metrics _follow_ | | Goodness of fit | |
| --- | --- | --- | --- | --- | --- | --- | --- |
|  |  | ρ1 | *P* | ρ2 | *P* | SRMR | CFI |
| **FPG** | **306** |  |  |  |  |  |  |
| PCGrimAA |  | 0.01 | 0.011 | 0.58 | 0.090 | 0.02 | 0.97 |
| PCPhenoAA |  | 0.01 | 0.917 | 0.23 | 0.773 | 0.02 | 0.98 |
| DunedinPACE |  | 0.17 | 0.197 | **0.04** | **0.001** | 0.02 | 0.98 |
| **HbA1c** | **312** |  |  |  |  |  |  |
| PCGrimAA |  | 0.01 | 0.061 | 0.69 | 0.203 | 0.02 | 0.97 |
| PCPhenoAA |  | <0.01 | 0.326 | 0.41 | 0.712 | 0.02 | 0.97 |
| DunedinPACE |  | 0.02 | 0.687 | **0.06** | **0.002** | 0.02 | 0.98 |
| **TyG** | **306** |  |  |  |  |  |  |
| PCGrimAA |  | 0.01 | 0.432 | **0.44** | **<0.001** | 0.02 | 0.97 |
| PCPhenoAA |  | <0.01 | 0.611 | 0.23 | 0.381 | 0.02 | 0.98 |
| DunedinPACE |  | 0.31 | 0.268 | **0.02** | **<0.001** | 0.02 | 0.98 |
| **T2DM** | **306** |  |  |  |  |  |  |
| PCGrimAA |  | >-0.01 | 0.582 | 0.56 | 0.047 | 0.02 | 0.96 |
| PCPhenoAA |  | <0.01 | 0.726 | 0.48 | 0.423 | 0.02 | 0.97 |
| DunedinPACE |  | 0.08 | 0.561 | 0.02 | 0.151 | 0.02 | 0.97 |

The ρ1 indicated the pathway from baseline epigenetic age metrics to phenotypes at follow-up, the ρ2 represented the pathway from baseline phenotypes to epigenetic age metrics;

Abbreviation: FPG, fasting plasma glucose; HbA1c, hemoglobin A1c; TyG, triglyceride glucose index; T2DM, Type 2 diabetes mellitus; PCGrimAA, PCGrimAge acceleration; PCPhenoAA, PCPhenoAge acceleration.

Significant results with *P <* 0.05 / (3 x 4) = 0.0042 were highlighted in bold font.

**Supplemental Table 8.** Cross-lagged relationships between epigenetic age metrics and T2DM or glycemic traits within MZ pairs

| Phenotypes | N | epigenetic age metrics _base_→ phenotypes _follow_ | | phenotypes _base_→ epigenetic age metrics _follow_ | | Goodness of fit | |
| --- | --- | --- | --- | --- | --- | --- | --- |
|  |  | ρ1 | *P* | ρ2 | *P* | SRMR | CFI |
| **FPG** | **92** |  |  |  |  |  |  |
| GrimAA |  | 0.01 | 0.466 | 0.62 | 0.403 | 0.01 | 1.00 |
| PhenoAA |  | -0.02 | 0.041 | 0.95 | 0.518 | 0.02 | 1.00 |
| DunedinPACE |  | -0.01 | 0.970 | <0.01 | 0.954 | 0.02 | 1.00 |
| **HbA1c** | **94** |  |  |  |  |  |  |
| GrimAA |  | <0.01 | 0.877 | 1.45 | 0.101 | 0.02 | 1.00 |
| PhenoAA |  | -0.01 | 0.067 | 3.25 | 0.034 | 0.02 | 1.00 |
| DunedinPACE |  | -0.22 | 0.258 | 0.01 | 0.730 | 0.02 | 1.00 |
| **TyG** | **92** |  |  |  |  |  |  |
| GrimAA |  | 0.04 | 0.231 | 0.61 | 0.034 | 0.02 | 1.00 |
| PhenoAA |  | -0.01 | 0.598 | 0.74 | 0.131 | 0.02 | 1.00 |
| DunedinPACE |  | 0.15 | 0.860 | 0.02 | 0.186 | 0.02 | 1.00 |

The ρ1 indicated the pathway from baseline epigenetic age metrics to phenotypes at follow-up, the ρ2 represented the pathway from baseline phenotypes to epigenetic age metrics;

Abbreviation: FPG, fasting plasma glucose; HbA1c, hemoglobin A1c; TyG, triglyceride glucose index; PCGrimAA, PCGrimAge acceleration; PCPhenoAA, PCPhenoAge acceleration.

Significant results with *P <* 0.05 / (3 x 3) = 0.0056 were highlighted in bold font.
